# Medicaid Coverage Withdrawal and Dispensing of Glucagon-Like Peptide-1 Receptor Agonists for Obesity

**DOI:** 10.7759/cureus.119224

**Published:** 2026-11-06

**Authors:** Fabrice T Nyambod, January G Msemakweli, Murishi Onesphore

**Affiliations:** 1 International Health, Johns Hopkins Bloomberg School of Public Health, Baltimore, USA; 2 Epidemiology, Johns Hopkins Bloomberg School of Public Health, Baltimore, USA; 3 Public Health, Johns Hopkins Bloomberg School of Public Health, Baltimore, USA

**Keywords:** antiobesity medications, difference-in-differences, drug utilization, glucagon-like peptide-1 receptor agonists, health policy, medicaid, obesity, semaglutide, tirzepatide

## Abstract

Introduction: Five state Medicaid programs withdrew coverage of glucagon-like peptide-1 receptor agonists (GLP-1 RAs) for obesity in late 2025 and January 2026, and others have narrowed coverage or are reconsidering it. The benefits of these drugs appear to depend on continued use, but whether withdrawal interrupts treatment for current users is unknown. This study examined changes in dispensing of obesity-indicated GLP-1 RAs after state Medicaid programs withdrew or restricted coverage.

Methods: This quasi-experimental study used quarterly Medicaid State Drug Utilization Data for 50 states and the District of Columbia from 2021 through the first quarter of 2026. Difference-in-differences and event-study models compared dispensing in states that withdrew coverage with dispensing in states that continued it, using each state's dispensing of diabetes-indicated GLP-1 RAs as a within-state comparison. The exposure was withdrawal or restriction of Medicaid coverage of GLP-1 RAs for obesity, identified from state agency documents. The main outcome was reimbursed prescriptions for obesity-indicated GLP-1 RAs per 1,000 enrollees per quarter. Estimates are reported with 95% confidence intervals (CIs). Data were analyzed from September to October 2026.

Results: The five withdrawing states accounted for 2,428,709 (65.2%) of 3,725,732 obesity-indicated GLP-1 RA prescriptions reimbursed by Medicaid in 2025. Compared with 12 continuing states, withdrawal was associated with an 84.7% decline in obesity-indicated dispensing (95% CI: 64.5%-93.4%; exact permutation p < 0.001), or 29.1 fewer prescriptions per 1,000 enrollees per quarter (95% CI: 14.3-43.9). No significant change was detected in diabetes-indicated dispensing (−3.2%; 95% CI: −23.4% to 22.2%), and differences between the two groups before the withdrawals were announced were small and not statistically significant. In the first quarter without coverage, obesity-indicated dispensing fell by 61% to 96% in individual withdrawing states. The four states that withdrew on January 1, 2026, reimbursed 661,353 obesity-indicated prescriptions in the preceding quarter and 134,647 in the next, a decline of 526,706 (79.6%). Dispensing of older antiobesity medications increased by at most 2.9 prescriptions per 1,000 enrollees. Narrower criteria in two states were followed by declines of 23%-37%.

Conclusions: In this quasi-experimental study, withdrawal of Medicaid coverage was associated with the loss of most reimbursed dispensing of GLP-1 RAs for obesity within one quarter, with little replacement by other agents. Protecting enrollees already receiving these drugs, for example, by honoring existing authorizations, might limit treatment interruption, but this has not been evaluated.

## Introduction

Glucagon-like peptide-1 receptor agonists (GLP-1 RAs) produce sustained weight loss in adults with obesity [1,2], and semaglutide reduced cardiovascular events in adults with overweight or obesity and established cardiovascular disease [3]. Responses to these drugs vary across patients [4]. Semaglutide (Wegovy) was approved for chronic weight management in June 2021 [5], and tirzepatide (Zepbound) was approved in November 2023 [6]. Obesity is common among Medicaid enrollees; 40% of adult enrollees had obesity in 2021 [7]. Whether Medicaid pays for these drugs, however, is left to each state. The Medicaid Drug Rebate Program requires states to cover nearly all approved outpatient drugs, including GLP-1 RAs prescribed for diabetes, but drugs used for weight loss are among the few classes that states may exclude [8].

States have used this discretion unevenly. By October 2025, 16 (31.4%) of the 51 state Medicaid programs covered GLP-1 RAs for obesity [9,10]. Studies comparing covering and noncovering states at single time points between 2022 and 2024 found higher use where the drugs were covered [11-13], use below 5% of eligible beneficiaries even where they were covered [12], and wide variation in prior authorization criteria [14]. Two recent analyses found that adopting coverage was associated with increased dispensing that continued to grow for two years or more [15,16].

Since late 2025, the direction has reversed. Under budget pressure, four programs ended adult coverage on January 1, 2026; North Carolina suspended coverage for 10 weeks beginning in October 2025; and other states narrowed clinical criteria or were considering restrictions [9,10]. Withdrawal presents states with a trade-off. Its savings come largely from ending reimbursement for people already receiving treatment, who often regain weight when these drugs are stopped [17]. The policy question therefore concerns not only how much a state saves but also how many current users lose treatment, how quickly they lose it, and whether they obtain it another way. None of the earlier studies could examine this question because no withdrawal had occurred within their study periods.

The objective of this study was to examine changes in Medicaid-reimbursed dispensing of GLP-1 RAs for obesity, and in gross Medicaid spending on these drugs, after state Medicaid programs withdrew coverage in late 2025 and January 2026 and after two states narrowed their coverage criteria. We compared states that withdrew coverage with states that continued it, using a difference-in-differences design. The same molecules are sold under separate brands for diabetes, which every state covered throughout the study period. Dispensing of these diabetes-indicated products in the same state and quarter therefore served as a comparison series for changes in enrollment, reporting, and general demand for GLP-1 RAs. Because these data count reimbursed prescriptions rather than individual patients, the study examined changes in reimbursed dispensing, not treatment discontinuation among individual enrollees.

## Materials and methods

Data sources

We measured dispensing with the Medicaid State Drug Utilization Data (SDUD), which report prescriptions and Medicaid reimbursement by state, quarter, and National Drug Code (NDC), separately for fee-for-service and managed care [18]. These data, which suppress cells with fewer than 11 prescriptions, have been used to measure state-level medication use, including the use of GLP-1 RAs [11,15,19]. Suppressed cells were counted as zero. The study period ran from the first quarter of 2021, before semaglutide was approved for weight management, through the first quarter of 2026, the most recent quarter available when data were obtained from the Centers for Medicare & Medicaid Services (CMS) in September 2026. Because the most recent quarter is subject to reporting lag, it is marked as provisional in all displays.

We classified products by NDC using the US Food and Drug Administration NDC Directory and the NDC Structured Product Labeling (SPL) Data Elements file [20]. Obesity-indicated GLP-1 RAs were semaglutide (Wegovy), tirzepatide (Zepbound), and liraglutide (Saxenda); diabetes-indicated GLP-1 RAs were semaglutide (Ozempic, Rybelsus), tirzepatide (Mounjaro), liraglutide (Victoza), dulaglutide, exenatide, and lixisenatide. Generic liraglutide carries one name and strength for both indications, so it was assigned to an indication by carton size, which differs between the reference products. Classification was by product label, because the SDUD do not record the indication for which a prescription was written. Wegovy is also indicated for cardiovascular risk reduction and Zepbound for obstructive sleep apnea, and since March and December 2024, respectively, states have been required to cover them for these indications even when they exclude drugs for weight loss [9]. Older antiobesity medications were phentermine, phentermine-topiramate, naltrexone-bupropion, orlistat, other sympathomimetics, and setmelanotide. Fixed-ratio combinations of a GLP-1 RA with insulin were excluded. We obtained monthly Medicaid enrollment by state from CMS and averaged it within each quarter [21]. We excluded Puerto Rico, which lacks an enrollment denominator.

Coverage policies

We compiled coverage events from state Medicaid bulletins, preferred drug lists, prior authorization criteria, and provider notices, supplemented by published trackers [9,10,22-27], and classified each as adoption, restriction (criteria narrowed without ending coverage), withdrawal, or reinstatement (Table 1). Two of the withdrawal notices gave fiscal reasons: California ended coverage under its enacted 2025-2026 state budget to reduce pharmacy spending [24], and North Carolina suspended coverage because of a state funding shortfall [22]. Table 1 lists the source document for each event. For each withdrawal, the last covered quarter was the last calendar quarter before the effective date, and the first uncovered quarter was the first calendar quarter fully under the new policy. A change effective on the first day of a quarter was assigned to that quarter (for example, January 1, 2026, to the first quarter of 2026), and a change effective later in a quarter was assigned to the next quarter. The same rule was applied to adoptions, restrictions, and reinstatements. North Carolina's suspension took effect on October 1, 2025, so its first uncovered quarter was the fourth quarter of 2025; coverage was reinstated on December 12, 2025, within that quarter, so the first quarter of 2026 was its first full quarter with coverage restored. Continuing states were those that covered GLP-1 RAs for obesity throughout the fourth quarter of 2025 and the first quarter of 2026; never-covering states were those with no coverage for obesity at any point. The reconstructed number of covering states matched KFF (formerly the Kaiser Family Foundation) counts for August 2024, October 2025, and January 2026 [9,10] and a published classification of formularies in early 2023 [11]. The analysis code is available from the corresponding author on request.

**Table 1:** State Medicaid coverage events for glucagon-like peptide-1 receptor agonists indicated for obesity (2021-2026) Coverage events were compiled from state Medicaid bulletins, preferred drug lists, prior authorization criteria, and provider notices [22-27], supplemented by published trackers [9,10]; the Source column names the document for each event, and coverage from product launch or by January 2023 was inferred from dispensing and published lists [9,11]; the Minnesota adoption date is from Khanna and colleagues [15]. The first quarter coded is the first calendar quarter fully under the new policy: the quarter of the effective date when the change took effect on the first day of a quarter, and otherwise the next quarter. Source documents for Tennessee, Mississippi, and Utah: Division of TennCare provider notice on obesity management agents (August 1, 2025); Mississippi Division of Medicaid prior authorization criteria for antiobesity select agents, version 1.3 (July 1, 2023); and Utah Department of Health and Human Services 2025 Medicaid statewide provider training, pharmacy program slides. BMI: body mass index; CDL: Contract Drugs List; EPSDT: Early and Periodic Screening, Diagnostic, and Treatment; FY: fiscal year; GLP-1: glucagon-like peptide-1; GS: General Session; KFF: formerly the Kaiser Family Foundation; MA Program: Pennsylvania Medical Assistance Program; MACE: major adverse cardiovascular events; MSA: Medical Services Administration; PA: prior authorization; PDL: preferred drug list; Q: quarter; QL: quantity limit; S.B.: Senate Bill; SDUD: State Drug Utilization Data; T2DM: type 2 diabetes mellitus; VAC: Virginia Administrative Code

State	Event	Effective date	First quarter coded	Policy detail	Source
California	Covered from product launch	Not documented	Q3 2021	Wegovy/Saxenda covered for weight loss from launch under Medi-Cal Rx CDL	Inferred from SDUD dispensing from the third quarter of 2021; prior coverage confirmed in the Medi-Cal Rx notice of October 21, 2025
California	Withdrew coverage	Jan 1, 2026	Q1 2026	Saxenda, Wegovy, Zepbound no longer covered for weight loss (adults 21+; EPSDT exception); PAs expired 2025-12-31	Medi-Cal Rx, Important update: GLP-1s for weight loss are not a covered benefit, October 21, 2025
Delaware	Covered from product launch	Not documented	Q3 2021	Covered with PA; adoption date not located	Inferred from SDUD dispensing; listed among covering states by KFF
Kansas	Covered from product launch	Not documented	Q3 2021	Covered with PA (severe obesity pathway); adoption date not located	Inferred from SDUD dispensing; listed among covering states by KFF
Massachusetts	Adopted coverage	Jan 1, 2024	Q1 2024	MassHealth began covering antiobesity agents; Wegovy and Saxenda preferred GLP-1s	MassHealth Pharmacy Facts 235, November 19, 2024, as described in published reporting; the primary document could not be retrieved
Massachusetts	Restricted clinical criteria	Jan 1, 2025	Q1 2025	Wegovy and Saxenda non-covered for overweight/obesity in adults; Zepbound preferred (from 2024-10-01)	MassHealth Pharmacy Facts 235, November 19, 2024, as described in published reporting; the primary document could not be retrieved
Massachusetts	Withdrew coverage	Jul 3, 2026	Q4 2026	All antiobesity GLP-1s non-covered for obesity-only use; Wegovy retained for MACE indication	MassHealth Pharmacy Facts 276, May 13, 2026, as described in published reporting; the primary document could not be retrieved
Michigan	Adopted coverage	Feb 1, 2022	Q2 2022	Pharmacy coverage of products exclusively for treatment of obesity with PA (bulletin MSA 21-49)	Michigan Department of Health and Human Services letter L 25-73, December 8, 2025, citing bulletin MSA 21-49
Michigan	Restricted clinical criteria	Jan 1, 2026	Q1 2026	GLP-1 for obesity limited to morbid obesity after failure of other interventions; existing PAs honored up to 6 months	Michigan Department of Health and Human Services letter L 25-73, December 8, 2025
Minnesota	Adopted coverage	Dec 1, 2021	Q1 2022	Antiobesity medications covered with PA from December 2021; 3-month Saxenda trial required before Wegovy	Minnesota Department of Human Services antiobesity prior authorization criteria (archived July 27, 2022), as cited by Khanna and colleagues
Missouri	Adopted coverage	Jan 1, 2025	Q1 2025	Zepbound covered for obesity with PA (Wegovy non-preferred, later excluded for weight management)	MO HealthNet preferred drug list effective February 1, 2025, and a provider newsletter, January 2025
Mississippi	Adopted coverage	Jul 1, 2023	Q3 2023	Anti-obesity select agents PA criteria v1.3: Wegovy and Saxenda preferred (age 12+), Contrave (18+)	Mississippi Division of Medicaid, prior authorization criteria for antiobesity select agents, version 1.3, July 1, 2023
North Carolina	Adopted coverage	Aug 1, 2024	Q4 2024	Coverage of obesity management medications (Saxenda, Wegovy age 12+; Zepbound 18+) with PA, $4 copay	North Carolina Medicaid announcement, July 17, 2024, and NCTracks prior authorization criteria, July 15, 2024
North Carolina	Withdrew coverage	Oct 1, 2025	Q4 2025	GLP-1 coverage for obesity discontinued due to state funding shortfall	North Carolina Medicaid announcement, September 5, 2025
North Carolina	Reinstated coverage	Dec 12, 2025	Q1 2026	Coverage reverted to criteria in place 2025-09-30; Wegovy, Zepbound, Saxenda back on PDL	North Carolina Medicaid announcement, December 19, 2025; reinstatement date also reported by KFF
New Hampshire	Covered by January 2023 (start date not documented)	Not documented	Q3 2021	Covered Saxenda/Wegovy/Zepbound for weight loss with PA; adoption date not located	Inferred from SDUD dispensing from the first quarter of 2023; listed as covering in early 2023 by Liu and Rome and in August 2024 by KFF
New Hampshire	Withdrew coverage	Jan 1, 2026	Q1 2026	GLP-1s not covered when prescribed solely for weight loss; other indications unchanged	New Hampshire Department of Health and Human Services provider notification, October 9, 2025
Pennsylvania	Adopted coverage	Jan 1, 2023	Q1 2023	MA Program began covering GLP-1 RAs for overweight/obesity with PA in January 2023	Pennsylvania Department of Human Services Medical Assistance Bulletin, November 24, 2025 (background section)
Pennsylvania	Withdrew coverage	Jan 1, 2026	Q1 2026	Coverage ends for GLP-1 RAs for overweight/obesity (adults 21+); non-GLP-1 obesity agents remain	Pennsylvania Department of Human Services Medical Assistance Bulletin, November 24, 2025, effective January 1, 2026
Rhode Island	Covered by January 2023 (start date not documented)	Not documented	Q3 2021	Covered with PA; adoption date not located	Inferred from SDUD dispensing from the first quarter of 2023; listed as covering in early 2023 by Liu and Rome and in August 2024 by KFF
South Carolina	Adopted coverage	Nov 1, 2024	Q1 2025	Wegovy, Saxenda (and generic) covered for obesity with PA (BMI, counseling, activity attestation)	Published reporting (KFF Health News, May 21, 2025; Milliman, March 19, 2026)
South Carolina	Withdrew coverage	Jan 1, 2026	Q1 2026	GLP-1s no longer covered for obesity/weight loss; no new PAs after 2025-12-01; PAs expire 2025-12-31	Published reporting (SC Daily Gazette, November 17, 2025) and a managed care plan member notice; the state bulletin could not be retrieved
Tennessee	Adopted coverage	Aug 1, 2025	Q4 2025	Obesity management agents covered for adults 21+ and children per label; Wegovy and Zepbound preferred with PA and QL	Division of TennCare provider notice on obesity management agents, August 1, 2025
Utah	Adopted coverage	Jul 1, 2025	Q3 2025	Wegovy, Saxenda, Zepbound covered for weight loss, fee-for-service members only, via S.B. 3 (2025 GS); funding limited to FY2026	Utah Department of Health and Human Services 2025 Medicaid statewide provider training, pharmacy program slides
Virginia	Covered from product launch	Not documented	Q3 2021	Weight-loss drugs covered with service authorization (12 VAC 30-50-210); Wegovy covered from launch under existing policy	Inferred from SDUD dispensing; the Virginia Department of Medical Assistance Services bulletin of June 23, 2023, refers to prior weight-loss drug criteria
Virginia	Restricted clinical criteria	Jul 1, 2023	Q3 2023	Service authorization tightened: BMI≥40 or ≥35 with 2+ chronic conditions, trial of non-GLP-1 agent, lifestyle attestation (2023 Appropriations Act Item 304.ZZZZ)	Virginia Department of Medical Assistance Services bulletin, Upcoming changes to service authorization criteria for weight-loss drugs, June 23, 2023
Virginia	Restricted clinical criteria	Jul 1, 2024	Q3 2024	Weight-loss drug coverage limited to BMI >40 or BMI >37 with hypertension, T2DM or dyslipidemia (2024 Appropriations Act); implemented August 2024	Virginia Department of Medical Assistance Services quarterly report RD520, September 5, 2024
Wisconsin	Covered from product launch	Not documented	Q3 2021	ForwardHealth antiobesity drug PA policy; Wegovy with PA, 12-month limit, two lifetime attempts	Inferred from SDUD dispensing; Wisconsin ForwardHealth antiobesity drug prior authorization policy

Outcomes

The primary outcome was prescriptions for obesity-indicated GLP-1 RAs per 1,000 Medicaid enrollees per state-quarter, summing fee-for-service and managed care. Secondary outcomes were the same measure for diabetes-indicated GLP-1 RAs, which served as the comparison series, and for older antiobesity medications, which could replace GLP-1 RAs after withdrawal. We also measured Medicaid reimbursement for obesity-indicated GLP-1 RAs before manufacturer rebates. Because federal and supplemental rebates are not reported in the SDUD, this measure reflects gross spending, not net savings to states.

Statistical analysis

We used a difference-in-differences design, comparing withdrawing states with continuing states before and after withdrawal. The comparison group comprised the 12 continuing states, including two that began coverage during 2025 (Tennessee and Utah) and one that restricted coverage in January 2026 (Michigan); all were retained in the primary analysis because each covered GLP-1 RAs for obesity in every post-withdrawal quarter, and they were excluded in sensitivity analyses. The analysis window ran from the first quarter of 2025 through the first quarter of 2026. It began after North Carolina and South Carolina had adopted coverage in late 2024. North Carolina's reinstated quarter was excluded. Every withdrawal was announced during the last covered quarter. The reference period was therefore the quarter before the announcement, and the announcement quarter was modeled with its own term to capture any anticipatory change. Models of the natural logarithm of one plus dispensing per 1,000 enrollees included state and quarter fixed effects, with estimates expressed as percentage changes, calculated as 100 × (exp(β) − 1). Each state-quarter was weighted equally. The absolute change in prescriptions per 1,000 enrollees was estimated separately by fitting the same model to untransformed dispensing per 1,000 enrollees. We fitted the same model to diabetes-indicated dispensing and also fitted a triple-difference model that used each state's diabetes-indicated series as a within-state control. Finally, an event-study model estimated changes by quarter relative to withdrawal; the pre-announcement quarters were tested jointly against zero. With five withdrawing states and two pre-announcement quarters, this test had limited power, so a nonsignificant result does not establish parallel trends; we therefore report each quarter's estimate with its 95% confidence interval (CI) and the smallest difference the test could detect with 80% power.

Because only five states withdrew coverage, inference was based on an exact permutation test. The observed withdrawal dates were assigned to every set of five of the 17 states, with North Carolina's October 2025 date given in turn to each state in the set (6,188 sets × 5 = 30,940 assignments, including the observed one). The model was reestimated for each assignment, and the P value was the proportion of assignments whose coefficient was at least as large in absolute value as the observed coefficient. The 95% CIs were calculated from standard errors clustered by state, using a t distribution with 16 degrees of freedom. Because cluster-robust standard errors can be too small when clusters are few, we also calculated wild cluster-restricted bootstrap P values and 95% CIs (Webb weights; 9,999 replications for P values and 1,999 for each value tested in the CI). For each withdrawing state, we also calculated the percentage change in obesity-indicated dispensing from the last covered to the first uncovered quarter, divided by the change in the state's diabetes-indicated dispensing over the same two quarters, so that a value of 1 means the two series moved together. North Carolina's reinstatement is reported as a single-state series. Restrictions were assessed with the same before-and-after comparison as withdrawals; these comparisons were descriptive and were not part of the difference-in-differences analysis.

We conducted seven sensitivity analyses. First, enrollees who knew that coverage would end might have filled prescriptions early, inflating the last covered quarter. To assess this, we measured each decline from the quarter before the withdrawal was announced and compared growth into the last covered quarter with growth in continuing states over the same quarters. Second, to assess whether incomplete reporting in the provisional quarter explained the declines, we examined North Carolina separately because its withdrawal took place in a fully reported quarter. Third, to assess whether the choice of window and reference quarter affected the estimate, we repeated the difference-in-differences analysis from the first quarter of 2024 with the last covered quarter as the reference, and estimated the event study over this longer window with California, New Hampshire, and Pennsylvania as the withdrawing states, because North Carolina and South Carolina began coverage during 2024. Fourth, to assess whether policy changes within the comparison group affected the estimate, we excluded Tennessee and Utah, Michigan, and all three. Fifth, because the diabetes-indicated series includes molecules without an obesity-indicated version (dulaglutide, exenatide, and lixisenatide), we restricted both series in the triple-difference model to semaglutide, tirzepatide, and liraglutide, and separately to semaglutide and tirzepatide. Sixth, because North Carolina's suspension lasted 10 weeks, we repeated the analysis with only the four sustained withdrawals. Seventh, we weighted state-quarters by Medicaid enrollment. To examine whether reporting lag differed by state or drug category, we compared each state's change in diabetes-indicated and older antiobesity dispensing from the fourth quarter of 2025 to the first quarter of 2026 with its change over the same quarters a year earlier. All tests were two-sided, with statistical significance set at p < 0.05. Data were analyzed from September to October 2026 using R version 4.6 (R Foundation for Statistical Computing, Vienna, Austria) with the fixest package.

Ethical considerations

Because the data were publicly available aggregates without individual identifiers, the study did not constitute human participants research, and institutional review board review and informed consent were not required. The study followed the Strengthening the Reporting of Observational Studies in Epidemiology (STROBE) reporting guideline [28].

## Results

State Medicaid programs

Of the 51 state programs (50 states and the District of Columbia), 17 (33.3%) covered at least one GLP-1 RA for obesity at some point during the study period, and 16 (31.4%) were covering in October 2025, after North Carolina's suspension (Table 1). Five (9.8%) withdrew coverage: North Carolina on October 1, 2025 (reinstated on December 12, 2025), and California, New Hampshire, Pennsylvania, and South Carolina on January 1, 2026. Twelve (23.5%) continued coverage through the first quarter of 2026, including Massachusetts, whose withdrawal took effect in July 2026. The remaining 34 (66.7%) never covered these agents. In 2025, the five withdrawing states enrolled 18.4 million (26.1%) of 70.5 million Medicaid beneficiaries and accounted for 2,428,709 (65.2%) of the 3,725,732 obesity-indicated GLP-1 RA prescriptions reimbursed by Medicaid (Table 2). Of the 96,062 obesity-indicated prescriptions in never-covering states, 62,127 (64.7%) were reimbursed by managed care plans, which may reimburse these agents without a statewide policy [11]; the remainder may reflect cardiovascular or sleep apnea indications [9]. Diabetes-indicated dispensing was similar across groups, with medians of 25.4, 27.3, and 25.9 prescriptions per 1,000 enrollees per quarter in withdrawing, continuing, and never-covering states, respectively.

**Table 2:** State Medicaid programs by coverage status for glucagon-like peptide-1 receptor agonists indicated for obesity (2025) Data are from the Medicaid State Drug Utilization Data [18] and Medicaid enrollment data [21]. Withdrawing states are California, New Hampshire, North Carolina, Pennsylvania, and South Carolina. Continuing states covered GLP-1 RAs for obesity throughout the fourth quarter of 2025 and the first quarter of 2026; they include Massachusetts, whose withdrawal took effect in July 2026, after the study period. Enrollment is the mean of quarterly enrollment in 2025, summed across states. Percentages are shares of the row total across the three groups; states include the District of Columbia. GLP-1: glucagon-like peptide-1; GLP-1 RA: glucagon-like peptide-1 receptor agonist

Characteristic	Withdrew coverage	Continued coverage	Never covered
States, N (%)	5 (9.8)	12 (23.5)	34 (66.7)
Medicaid enrollment, millions (%)	18.4 (26.1)	11.4 (16.1)	40.8 (57.8)
Obesity-indicated GLP-1 RA prescriptions per 1,000 enrollees per quarter, median (range)	30.1 (3.1-41.3)	25.9 (0.1-42.3)	0.5 (0.0-3.4)
Diabetes-indicated GLP-1 RA prescriptions per 1,000 enrollees per quarter, median (range)	25.4 (14.7-35.0)	27.3 (13.2-35.4)	25.9 (8.1-55.1)
Obesity-indicated GLP-1 RA prescriptions, N (%)	2,428,709 (65.2)	1,200,961 (32.2)	96,062 (2.6)

Withdrawal of coverage

Before withdrawal, dispensing rose steeply in the withdrawing states. In California, New Hampshire, and Pennsylvania, obesity-indicated dispensing increased 6.4- to 9.0-fold from the first quarter of 2024 to the last covered quarter. North Carolina and South Carolina began coverage during 2024 (Figure 1). Before the withdrawals were announced, event-study estimates for withdrawing relative to continuing states were −0.6% (95% CI: −36.4% to 55.5%) three quarters before the announcement and −3.8% (95% CI: −22.5% to 19.4%) two quarters before (joint p = 0.85; Figure 2). With 80% power, the test could detect differences of about 36% or more two quarters before and 88% or more three quarters before the announcement. Relative to continuing states, withdrawal was associated with an 84.7% decline in obesity-indicated dispensing (95% CI: 64.5%-93.4%; exact p < 0.001; wild bootstrap 95% CI: 58.5%-94.0%; p < 0.001), or 29.1 fewer prescriptions per 1,000 enrollees per quarter (95% CI: 14.3-43.9). In the announcement quarter, dispensing changed by −10.0% (95% CI: −33.9% to 22.5%). No significant change was detected in diabetes-indicated dispensing (−3.2%; 95% CI: −23.4% to 22.2%; wild bootstrap p = 0.82), although the interval does not exclude moderate changes in either direction. The triple-difference estimate was −84.2% (95% CI: −92.0% to −68.9%; exact p < 0.001).

**Figure 1:**
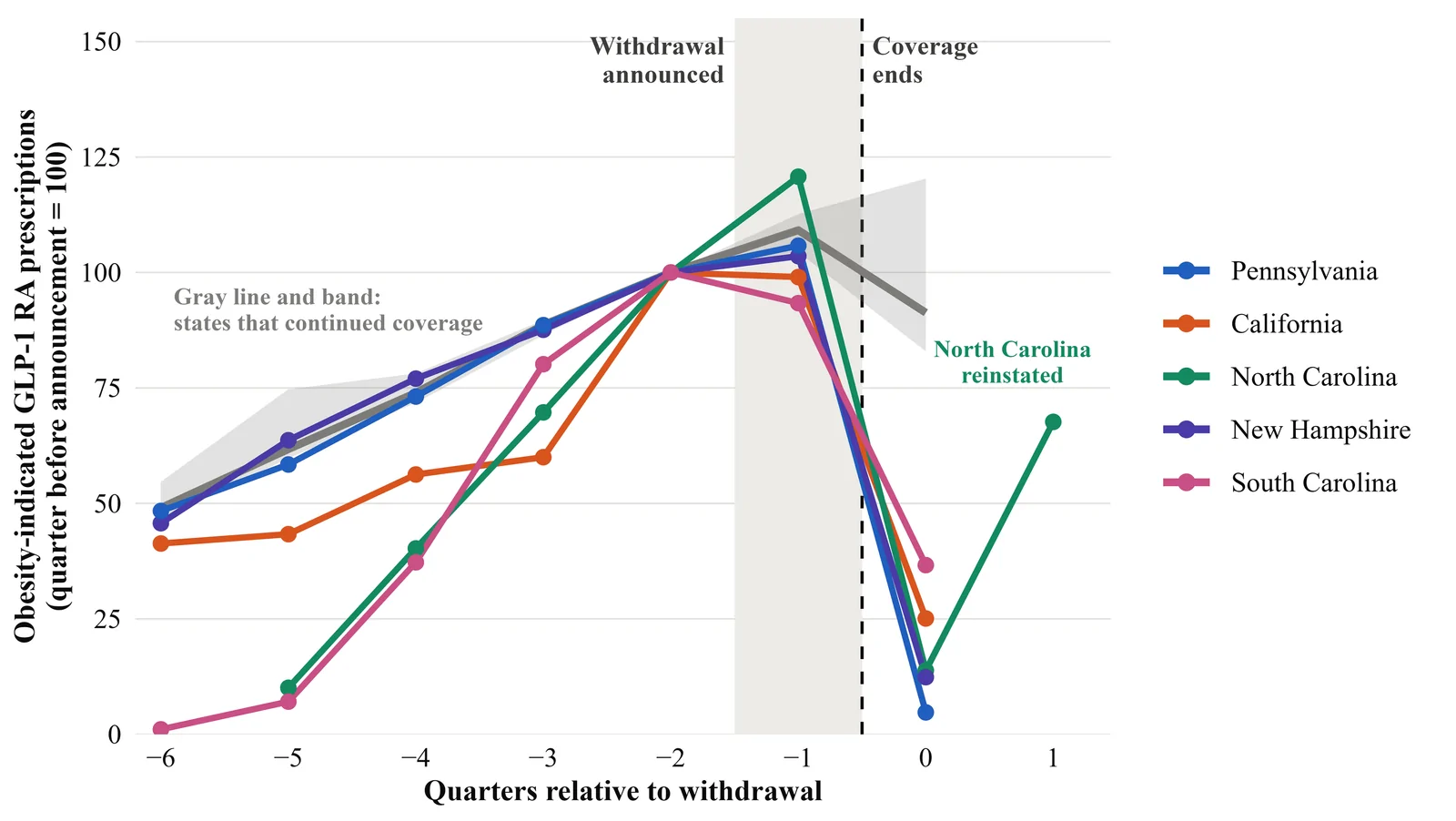
Obesity-indicated glucagon-like peptide-1 receptor agonist dispensing in states that withdrew Medicaid coverage Prescriptions per 1,000 enrollees in each withdrawing state by quarter relative to withdrawal, indexed to the quarter before the withdrawal was announced (= 100). The gray line and band show the median and interquartile range of states that continued coverage over the same calendar quarters. North Carolina's coverage was reinstated on December 12, 2025, during its first uncovered quarter. The shaded quarter marks the announcement, and the dashed line marks the end of coverage. The figure was created with R version 4.6 (R Foundation for Statistical Computing, Vienna, Austria) using the ggplot2 package. GLP-1: glucagon-like peptide-1; GLP-1 RA: glucagon-like peptide-1 receptor agonist

**Figure 2:**
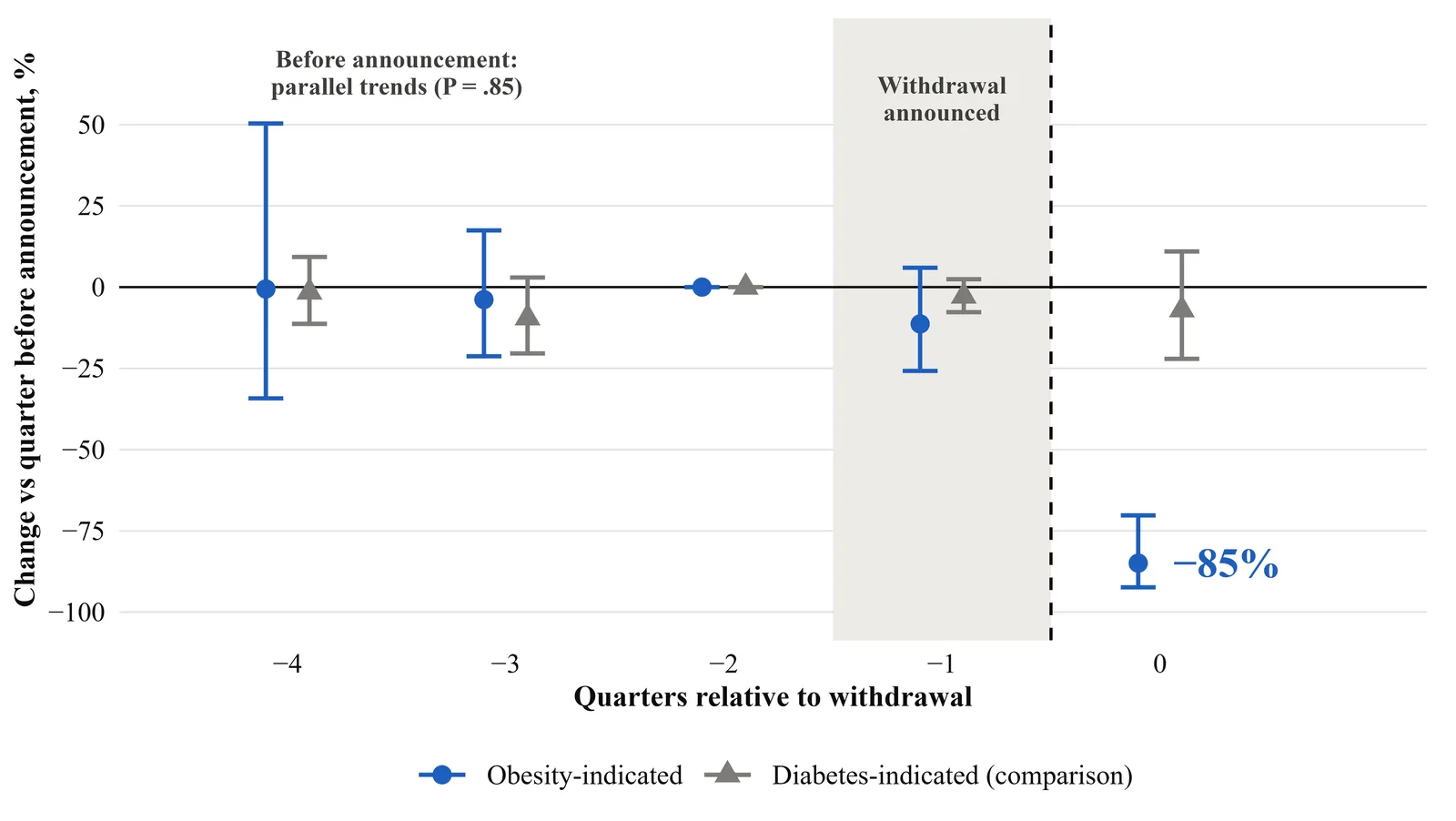
Change in glucagon-like peptide-1 receptor agonist dispensing after withdrawal of Medicaid coverage, relative to continuing states Event-study estimates of the percentage change in obesity-indicated and diabetes-indicated dispensing in withdrawing states relative to continuing states, compared with the quarter before the announcement, from models with state and quarter fixed effects; error bars indicate 95% CIs. The P value is from a joint test of the two quarters before the announcement. The shaded quarter marks the announcement, and the dashed line marks the end of coverage. The figure was created with R version 4.6 (R Foundation for Statistical Computing, Vienna, Austria) using the ggplot2 package. CI: confidence interval; GLP-1: glucagon-like peptide-1; GLP-1 RA: glucagon-like peptide-1 receptor agonist

In the first quarter without coverage, declines in obesity-indicated dispensing ranged from 60.8% in South Carolina to 95.5% in Pennsylvania, where dispensing went from 47.5 to 2.1 per 1,000 enrollees (Table 3). Over the same quarters, diabetes-indicated dispensing in these states changed by −35.0% to 7.8%. Among the 12 continuing states, the median change in obesity-indicated dispensing was −0.4% (range: −42.6% to 215.5%).

**Table 3:** Obesity-indicated glucagon-like peptide-1 receptor agonist dispensing per 1,000 Medicaid enrollees before and after coverage withdrawal, by state Data are from the Medicaid State Drug Utilization Data [18] and Medicaid enrollment data [21]. The last covered and first uncovered quarters are the third and fourth quarters of 2025 for North Carolina and the fourth quarter of 2025 and the first quarter of 2026 for the other states; continuing states are compared over the fourth quarter of 2025 and the first quarter of 2026. The change relative to diabetes-indicated change is the ratio of obesity-indicated dispensing in the first uncovered quarter to the last covered quarter, divided by the same ratio for diabetes-indicated dispensing; a value of 1 indicates that the two series changed proportionally. The first quarter of 2026 is provisional.

State	Date coverage ended	Obesity-indicated, last covered quarter	Obesity-indicated, first quarter without coverage	Obesity-indicated change, %	Diabetes-indicated change, %	Obesity-indicated change relative to diabetes-indicated change	Older antiobesity medications, change per 1,000
Pennsylvania	Jan 1, 2026	47.5	2.1	−95.5	−35.0	0.07	1.9
North Carolina	Oct 1, 2025	35.9	4.1	−88.5	1.6	0.11	2.9
New Hampshire	Jan 1, 2026	33.8	4.0	−88.0	−11.4	0.14	−0.1
California	Jan 1, 2026	45.0	11.4	−74.7	7.8	0.24	0.2
South Carolina	Jan 1, 2026	3.8	1.5	−60.8	7.3	0.37	0.0
Continued coverage (n = 12), median (range)	Not applicable	29.1 (0.4-53.1)	23.5 (1.2-67.3)	−0.4 (−42.6 to 215.5)	−1.4 (−40.5 to 18.9)	1.05 (0.61 to 3.20)	−0.1 (−1.2 to 0.6)

Relative to the diabetes-indicated series, obesity-indicated dispensing in withdrawing states changed by factors of 0.07-0.37, compared with a median of 1.05 (range: 0.61-3.20) in continuing states (Table 3). Across the four states that withdrew on January 1, 2026, obesity-indicated prescriptions fell from 661,353 in the last covered quarter to 134,647 in the next, a decline of 526,706 (79.6%), while diabetes-indicated prescriptions fell from 474,255 to 452,216, a decline of 22,039 (4.6%). Across all five withdrawing states, Medicaid reimbursement for obesity-indicated GLP-1 RAs went from $911.0 million in the last covered quarter to $172.4 million in the first quarter without coverage, a decline of $738.6 million (81.1%) before rebates, which were not observed; net savings to states would have been smaller.

Dispensing of older antiobesity medications changed by −0.1 to 2.9 per 1,000 enrollees in withdrawing states. The largest increase, 2.9 per 1,000 in North Carolina, compared with a decline of 31.8 per 1,000 in obesity-indicated GLP-1 RA dispensing in that state. In North Carolina, obesity-indicated dispensing was 35.9 per 1,000 in its last covered quarter; 4.1 in the fourth quarter of 2025, when coverage was suspended until December 12; and 20.1 in the first full quarter after reinstatement.

Median growth into the last covered quarter was 3.5% in withdrawing states, compared with 10.2% in continuing states over the same quarters, although growth varied across withdrawing states, from −6.6% in South Carolina to 20.7% in North Carolina. Declines measured from the quarter before each announcement ranged from 63.4% to 95.2% (Table 4). North Carolina's decline of 88.5% occurred in a quarter that was not provisional. With the analysis starting in the first quarter of 2024 and the last covered quarter as the reference, the estimated decline was 79.8% (95% CI: 53.6%-91.2%; exact p = 0.002). Over this longer window, with California, New Hampshire, and Pennsylvania as the withdrawing states, the quarterly pre-announcement estimates ranged from −24.7% to 8.3%, and none was statistically significant on its own, but the joint test rejected equal pre-announcement trends (p = 0.004); the most negative estimates were in the first half of 2024, when dispensing in these states grew faster than in continuing states (Figure 1). In the first quarter without coverage, the estimated decline in these three states was 88.2% (95% CI: 72.6%-94.9%). In the other sensitivity analyses, the estimated decline ranged from 81.3% to 85.2% (Table 5): 82.2% (95% CI: 58.2%-92.4%) without Tennessee, Utah, and Michigan; 81.8% (95% CI: 63.3%-91.0%) in the triple-difference model restricted to shared molecules; 83.7% (95% CI: 54.5%-94.1%) with only the four sustained withdrawals; and 82.8% (95% CI: 61.6%-92.3%) with enrollment weights. From the fourth quarter of 2025 to the first quarter of 2026, diabetes-indicated dispensing changed by −40.5% to 18.9% across the 17 states, compared with −21.1% to 10.2% over the same quarters a year earlier.

**Table 4:** Anticipation check: obesity-indicated glucagon-like peptide-1 receptor agonist dispensing per 1,000 Medicaid enrollees around coverage withdrawal Data are from the Medicaid State Drug Utilization Data [18] and Medicaid enrollment data [21]. Values are prescriptions per 1,000 enrollees per quarter. The quarter before announcement is the last quarter before each withdrawal was announced. Growth into the last covered quarter would indicate prescriptions filled early in anticipation of withdrawal. The first quarter of 2026 is provisional.

State	Quarter before announcement	Last covered quarter	First quarter without coverage	Change into last covered quarter, %	Decline from last covered quarter, %	Decline from quarter before announcement, %
California	45.5	45.0	11.4	−1.0	−74.7	−74.9
North Carolina	29.7	35.9	4.1	20.7	−88.5	−86.2
New Hampshire	32.7	33.8	4.0	3.5	−88.0	−87.6
Pennsylvania	44.9	47.5	2.1	5.8	−95.5	−95.2
South Carolina	4.0	3.8	1.5	−6.6	−60.8	−63.4

**Table 5:** Sensitivity analyses of the change in obesity-indicated glucagon-like peptide-1 receptor agonist dispensing after withdrawal of Medicaid coverage Data are from the Medicaid State Drug Utilization Data [18] and Medicaid enrollment data [21]. Estimates are percentage changes in obesity-indicated dispensing in withdrawing relative to continuing states, from models of the natural logarithm of one plus dispensing per 1,000 enrollees with state and quarter fixed effects, fitted to data from the first quarter of 2025 through the first quarter of 2026. Triple-difference models use each state's diabetes-indicated series, restricted as shown, as a within-state control. The 95% CIs are from standard errors clustered by state, except for the wild cluster bootstrap row, which inverts the wild cluster restricted bootstrap test (Webb weights). The first quarter of 2026 is provisional. CI: confidence interval; DiD: difference-in-differences; GLP-1 RA: glucagon-like peptide-1 receptor agonist

Analysis	States, n	Estimate, % (95% CI)
Primary analysis, obesity-indicated	17	−84.7 (−93.4 to −64.5)
Primary analysis, wild cluster bootstrap CI	17	−84.7 (−94.0 to −58.5)
Excluding Tennessee and Utah	15	−81.8 (−92.0 to −58.3)
Excluding Michigan	16	−85.2 (−93.8 to −64.9)
Excluding Tennessee, Utah, and Michigan	14	−82.2 (−92.4 to −58.2)
Four sustained withdrawals (North Carolina excluded)	16	−83.7 (−94.1 to −54.5)
Weighted by Medicaid enrollment	17	−82.8 (−92.3 to −61.6)
Triple difference, all diabetes-indicated GLP-1 RAs	17	−84.2 (−92.0 to −68.9)
Triple difference, semaglutide, tirzepatide, and liraglutide	17	−81.8 (−91.0 to −63.3)
Triple difference, semaglutide and tirzepatide	17	−81.3 (−90.8 to −62.0)
Triple difference, four sustained withdrawals	16	−83.0 (−92.3 to −62.3)

Restrictions

After Virginia narrowed its criteria in mid-2023, obesity-indicated dispensing fell from 4.8 to 3.0 per 1,000 enrollees over two quarters (−37%); after a second restriction in 2024, it fell from 9.1 to 7.0 (−23%). In both instances, dispensing rose again within a year. After Michigan restricted coverage to morbid obesity in January 2026, with existing authorizations honored for six months, dispensing fell from 49.4 to 37.7 per 1,000 (−24%), while diabetes-indicated dispensing changed by 3.1%.

## Discussion

In this quasi-experimental study of Medicaid dispensing in all states through the first quarter of 2026, withdrawal of coverage for GLP-1 RAs for obesity was associated with a decline of about 85% in reimbursed dispensing within one quarter. Dispensing of older antiobesity medications and of the diabetes-labeled versions of the same drugs did not rise enough to offset the decline. These aggregate data cannot, however, exclude substitution by some enrollees. Narrower criteria were followed by smaller declines.

These findings suggest that abrupt withdrawal of coverage ended most Medicaid-reimbursed dispensing of these drugs. Whether the decline reflected discontinuation by current users, fewer new users, or a shift to other sources of payment cannot be determined from aggregate data and requires patient-level data. In the five withdrawing states, the first quarter without coverage was accompanied by 607,145 (80.7%) fewer prescriptions and $738.6 million (81.1%) less in gross reimbursement; savings net of rebates, which were not observed, would have been smaller. Because the benefits of these drugs appear to depend on continued use [17], any savings may involve clinical trade-offs, which this study did not measure. States facing budget pressure may have options that limit treatment interruption. Narrower criteria in Virginia and Michigan were followed by declines of 23%-37%, and Michigan honored existing authorizations for six months when it restricted coverage [26]. These are descriptive before-and-after comparisons, and Michigan honored existing authorizations at the same time as it narrowed eligibility, so the separate effect of honoring authorizations cannot be estimated from these data. Protecting enrollees already receiving treatment, through grandfathered authorizations or a phased withdrawal, might allow states to reduce spending on new use without interrupting treatment for current users; whether it does, and at what cost to net savings, has not been tested.

To our knowledge, no earlier study has examined what happened when Medicaid stopped paying for these agents. Khanna et al. and Wing et al. described dispensing that built up over two years or more after coverage began; in the states studied here, most of that volume was lost within three months of withdrawal [15,16]. Dun et al. described coverage as necessary but not sufficient for use [12], and our data bear mainly on the first half of that statement. Pennsylvania, for example, went from one of the highest dispensing rates in the country to 2.1 per 1,000 enrollees, a rate lower than that in some states that had never covered the drugs.

A decline of this size is broadly consistent with patient-level evidence. Most formulary rejections of branded drugs end with no fill within 90 days [29], and loss of coverage and cost are among the reasons people most often give for stopping a GLP-1 RA [30].

Where these enrollees went cannot be established from aggregate data. Older antiobesity medications accounted for less than a 10th of the lost volume. The diabetes-labeled products are the more obvious alternative, and evidence on switching between labels after adoption is mixed [15,16]. Had switching been common after withdrawal, diabetes-indicated dispensing would have risen; it rose by 8% at most and fell in Pennsylvania and New Hampshire. Because the diabetes-indicated estimate was imprecise, however, the absence of a detectable increase does not rule out switching by some enrollees. Some enrollees may have paid out of pocket or used compounded products. North Carolina's experience suggests that the loss may not be fully reversed in the short term: after coverage was restored, dispensing returned to about half of its earlier level in the next full quarter.

One potential complication is that the first quarter of 2026 was provisional and incompletely reported at the time of analysis. Several continuing states also had lower dispensing in that quarter, in some cases alongside lower diabetes-indicated dispensing, which is the pattern incomplete reporting would produce. Changes in diabetes-indicated dispensing into this quarter also varied more across states than a year earlier, which suggests that completeness differed by state. The triple-difference model compares each state's obesity-indicated series with its own diabetes-indicated series and is therefore less sensitive to such differences, provided that completeness was similar across drug categories within a state. However, no continuing state came close to the relative declines in the withdrawing states, and North Carolina, whose withdrawal fell in a fully reported quarter, declined by a similar amount. Unlike the other withdrawals, North Carolina's suspension lasted only 10 weeks; excluding it changed the pooled estimate little (Table 5). We also found no consistent evidence of substantial early filling once withdrawals had been announced, although growth into the last covered quarter varied across states and was highest in North Carolina.

Limitations

This study has several limitations. First, the data are aggregated by state and quarter, so person-level discontinuation, switching to a diabetes-indicated product, out-of-pocket purchase, and continuation under the federal exception for enrollees younger than 21 years could not be observed. Second, prescriptions do not measure the number of people treated, because days' supply per prescription was not available. Third, the withdrawal estimates rest on five states and, for four of them, on the most recent SDUD quarter, which is subject to reporting lag, and follow-up after each withdrawal was limited to one quarter; the estimates should be updated as later quarters are released. Fourth, the diabetes-indicated series is a valid comparison only if substitution between labels is small, and evidence on substitution is mixed [15,16]. In addition, products were classified by label, not by the indication recorded for each prescription (Table 6). Fifth, the difference-in-differences design assumes that continuing states represent the trend withdrawing states would otherwise have followed; pre-announcement estimates in the primary window were small and not statistically significant, but the test had limited power, dispensing grew faster in withdrawing states during 2024, and states that chose to withdraw may differ in ways that this design cannot capture. The comparison group also included Michigan, which restricted coverage in January 2026, and two states that began coverage in 2025; excluding them changed the estimate little (Table 5). Sixth, managed care plans may reimburse these agents without a statewide policy, and coverage dates for states that covered from product launch were inferred from dispensing and published lists. Seventh, the withdrawing states included California and Pennsylvania, two of the largest Medicaid programs, and each withdrawing state ended coverage on a single date; the findings may not apply to smaller programs or to withdrawals phased in over time. Eighth, suppression of cells with fewer than 11 prescriptions affected many state-quarters but is unlikely to have altered the conclusions, given the size of the changes. Ninth, reimbursement was measured before rebates, and clinical outcomes were not observed, so neither net savings nor clinical harms could be estimated. Tenth, the study did not assess the effectiveness, safety, or cost-effectiveness of these drugs, which bear on whether Medicaid programs should cover them.

**Table 6:** National Drug Codes assigned to each medication group Codes are from the US Food and Drug Administration NDC Directory and Structured Product Labeling Data Elements file [20] and are those with at least one Medicaid prescription in the State Drug Utilization Data [18] from the first quarter of 2021 through the first quarter of 2026. Generic liraglutide was assigned to an indication by carton size. Fixed-ratio combinations of a GLP-1 RA with insulin were excluded. This table is the authors' original compilation. GLP-1 RA: glucagon-like peptide-1 receptor agonist; NDC: National Drug Code

Series	Molecule (brand)	NDCs, n	National Drug Codes (11-digit)
Obesity-indicated GLP-1 RA	Semaglutide (Wegovy)	9	00169-4404-31, 00169-4409-31, 00169-4415-31, 00169-4425-31, 00169-4501-14, 00169-4505-14, 00169-4517-14, 00169-4524-14, 00169-4525-14
Obesity-indicated GLP-1 RA	Tirzepatide (Zepbound)	12	00002-2457-01, 00002-2457-80, 00002-2460-01, 00002-2460-80, 00002-2471-01, 00002-2471-80, 00002-2484-01, 00002-2484-80, 00002-2495-01, 00002-2495-80, 00002-2506-01, 00002-2506-80
Obesity-indicated GLP-1 RA	Liraglutide (Saxenda and generic)	3	00169-2800-15, 00480-7250-46, 69097-0910-55
Diabetes-indicated GLP-1 RA	Semaglutide (Ozempic, Rybelsus)	17	00169-4130-01, 00169-4130-13, 00169-4132-11, 00169-4132-12, 00169-4136-02, 00169-4136-11, 00169-4181-03, 00169-4181-13, 00169-4181-97, 00169-4303-13, 00169-4303-30, 00169-4307-13, 00169-4307-30, 00169-4314-13, 00169-4314-30, 00169-4772-11, 00169-4772-12
Diabetes-indicated GLP-1 RA	Tirzepatide (Mounjaro)	12	00002-1457-01, 00002-1457-80, 00002-1460-01, 00002-1460-80, 00002-1471-01, 00002-1471-80, 00002-1484-01, 00002-1484-80, 00002-1495-01, 00002-1495-80, 00002-1506-01, 00002-1506-80
Diabetes-indicated GLP-1 RA	Liraglutide (Victoza and generic)	10	00143-9144-02, 00143-9144-03, 00169-4060-12, 00169-4060-13, 00480-3667-20, 00480-3667-22, 70748-0346-02, 70748-0346-03, 71288-0563-84, 71288-0563-85
Diabetes-indicated GLP-1 RA	Dulaglutide	8	00002-1433-01, 00002-1433-80, 00002-1434-01, 00002-1434-80, 00002-2236-01, 00002-2236-80, 00002-3182-01, 00002-3182-80
Diabetes-indicated GLP-1 RA	Exenatide	8	00310-6512-01, 00310-6524-01, 00310-6530-01, 00310-6530-04, 00310-6540-01, 00310-6540-04, 70121-1685-01, 70121-1686-01
Diabetes-indicated GLP-1 RA	Lixisenatide	2	00024-5745-02, 00024-5747-02
Older antiobesity medication	Phentermine	40	00527-1308-01, 00527-1445-01, 00527-1445-10, 10702-0001-09, 10702-0025-01, 10702-0025-03, 10702-0025-10, 10702-0026-01, 10702-0026-10, 10702-0027-01, 10702-0027-10, 10702-0028-01, 10702-0028-10, 10702-0029-01, 10702-0029-03, 10702-0029-10, 11534-0157-01, 11534-0157-03, 11534-0159-01, 11534-0159-03, 11534-0160-01, 11534-0160-03, 11534-0160-30, 11534-0176-01, 11534-0176-03, 13107-0061-01, 13107-0061-99, 43547-0404-10, 51224-0101-50, 51224-0101-70, 51224-0202-50, 51224-0202-70, 51224-0203-50, 51224-0203-70, 53489-0676-01, 53489-0676-10, 64850-0810-01, 64850-0810-10, 68094-0802-50, 68094-0910-50
Older antiobesity medication	Phentermine-topiramate	13	00480-2299-56, 00480-3295-56, 00480-3296-56, 00480-3297-56, 43598-0624-30, 43598-0625-30, 62541-0201-30, 62541-0202-30, 62541-0203-30, 62541-0204-30, 66993-0780-30, 66993-0781-30, 66993-0782-30
Older antiobesity medication	Naltrexone-bupropion	1	51267-0890-99
Older antiobesity medication	Orlistat	2	61269-0460-90, 61269-0565-90
Older antiobesity medication	Other sympathomimetics (benzphetamine, diethylpropion, phendimetrazine)	9	00527-1475-01, 00527-1477-01, 10702-0040-03, 10702-0044-01, 10702-0077-01, 64850-0440-01, 69543-0409-30, 69543-0410-10, 72989-0409-30
Older antiobesity medication	Setmelanotide	1	72829-0010-01

## Conclusions

In this quasi-experimental study, withdrawal of state Medicaid coverage for GLP-1 RAs indicated for obesity was associated with the loss of most reimbursed dispensing within one quarter, with little replacement by older antiobesity agents and no detectable change in the same molecules prescribed for diabetes. Because the benefits of these drugs may diminish when treatment stops, protecting enrollees already receiving treatment is a policy option for states that reduce coverage, and its effects warrant evaluation. Later quarters of utilization data and person-level data could show whether these declines persisted and whether enrollees who lost coverage obtained treatment by other means.
